# Prophylactic Versus Reactive Ventricular Tachycardia Ablation in Repaired Tetralogy of Fallot: A Narrative Review

**DOI:** 10.3390/jcdd13070299

**Published:** 2026-07-01

**Authors:** Zahra Yousefli, Jonathan Chrispin, Ari Cedars, Stacy Fisher, Glenn T. Wetzel, Konstantinos N. Aronis

**Affiliations:** 1 Division of Cardiology, Department of Medicine, Johns Hopkins Hospital School of Medicine, Baltimore, MD 21287, USA; 2 Division of Pediatric Cardiology, Department of Pediatrics, Johns Hopkins School of Medicine, Baltimore, MD 21287, USA; 3 Helen B. Taussig Heart Center, Department of Pediatrics, Johns Hopkins Hospital, Baltimore, MD 21287, USA

**Keywords:** tetralogy of Fallot, ventricular tachycardia, catheter ablation, slowly conducting anatomical isthmus, electroanatomical mapping, adult congenital heart disease

## Abstract

Ventricular tachycardia and sudden cardiac death remain the principal late causes of mortality in repaired tetralogy of Fallot. Clinical practice is evolving from a “reactive” paradigm centered on defibrillator therapy and post-event ablation toward a “proactive” paradigm targeting slowly conducting anatomical isthmuses before clinical arrhythmias become manifest. Monomorphic ventricular tachycardia in this population typically occurs due to a discrete, anatomically defined set of slowly conducting isthmuses bounded by surgical patches or incisions and valve annuli. Substrate-targeted catheter and surgical ablation are technically feasible, safe, and associated with high arrhythmia-free survival when complete bidirectional block is achieved. The current indication for “proactive” ablation is for substrate evaluation before transcatheter pulmonary valve replacement, after which endocardial access to the dominant isthmus may be permanently obscured. Pulmonary valve replacement alone does not abolish the arrhythmogenic substrate, thus providing the rationale for combining valve intervention with proactive ablation. This narrative review discusses substrate biology, risk stratification, comparative outcomes of reactive and proactive ablation strategies, and the role of pulmonary valve replacement. It also proposes an operational pathway integrating both approaches within shared decision-making. The ongoing CATAPULT-TOF study and subsequent multicenter work will determine the populations in which proactive substrate evaluation should become routine.

## 1. Introduction

### 1.1. The Problem: SCD After TOF Repair

Tetralogy of Fallot (TOF) is the most common cyanotic congenital heart disease and surgical advances over the past decades have allowed more than 90% of patients to achieve long-term survival [1]. However, nearly 25% of patients with repaired tetralogy of Fallot (rTOF) face risks of life-threatening macro-reentrant ventricular tachycardia (VT) and sudden cardiac death (SCD) [2,3,4], with an annual SCD incidence of 0.9 to 2% [2,5]. In patients with rTOF, the primary arrhythmogenic substrate consists of slowly conducting anatomic isthmuses (SCAIs) formed by surgical scars or patches, and the pulmonary or tricuspid valve annuli [3]. Conduction delay, residual shunts, and acquired scarring from surgery contribute to evolving VT substrate as patients age, leading to lower conduction velocity and higher VT risk [6,7,8].

### 1.2. An Evolving Therapeutic Landscape

Two complementary pillars currently form the foundation of VT management in rTOF: (1) implantable cardioverter-defibrillator (ICD) therapy for primary or secondary prevention of SCD [9], and (2) catheter ablation of the arrhythmogenic substrate [8,10,11]. Historically, catheter ablation has been offered after a documented arrhythmic event [11,12]. Advances in substrate characterization, electroanatomic mapping (EAM), and an improved understanding of the substrate and its evolution have raised the possibility of intervening before the first episode of clinical VT [8,13,14]. A parallel motivation has emerged in patients undergoing transcatheter pulmonary valve replacement: deployment of a valve across the native right ventricular outflow tract can render the dominant arrhythmogenic substrate inaccessible to subsequent ablation, thus providing an additional rationale for earlier substrate evaluation [7]. Whether this shift to a primary prevention strategy is justified by existing data, and in which patient subgroups it should be applied, remain central unresolved questions that motivate the present narrative review.

### 1.3. Scope and Definitions

This narrative review synthesizes current evidence on reactive and proactive (prophylactic) catheter ablation of VT in adults and adolescents with rTOF, with attention to substrate characterization, peri-PVR timing, ICD decision-making, and outstanding gaps in evidence. Building on the 2022 AHA Scientific Statement on arrhythmias in rTOF [3] and the 2021 JACC review on preventing arrhythmic death in TOF [15], the present narrative review focuses specifically on the operational integration of proactive and reactive substrate ablation. It also proposes a decision framework that translates the substrate biology and the emerging proactive evidence into a clinical pathway. For the purposes of this review, reactive VT ablation is defined as substrate- or VT-targeted catheter ablation performed in response to documented sustained VT, VT storm, or recurrent appropriate ICD therapies [10]. Proactive ablation is defined as substrate-based EAM and ablation of arrhythmogenic SCAI performed before any documented sustained VT, typically at the time of a planned cardiac procedure or in selected asymptomatic patients with high-risk substrate features, or inducible VT in electrophysiology study (EPS). Risk stratification for primary-prevention ICD implantation, while inseparable from the proactive strategy, is addressed only insofar as it impacts ablation decision-making.

Given the arrhythmogenic substrate in rTOF matures over time [6], pediatric patients often have immature or incompletely developed slowly conducting isthmuses, whereas adult patients have accumulated decades of remodeling, fibrosis, and hemodynamic exposure; therefore, the pediatric population is outside the scope of this review and evidence derived from adult or adolescent cohorts should not be extrapolated directly to younger children.

## 2. Methods

In this narrative review we searched PubMed, Google Scholar and ClinicalTrials.gov from inception through April 2026 for studies on VT ablation in rTOF, using combinations of terms like ‘repaired tetralogy of Fallot’, ‘ventricular tachycardia’, ‘ablation’, ‘substrate’, ‘prophylactic’. Reference lists of identified studies and relevant guideline documents were hand-searched to capture additional sources. Eligibility was confined to English-language original studies, multicenter cohorts, registries, prospective trial protocols, scientific statements, guideline documents, and prior narrative or systematic reviews directly addressing ventricular tachycardia substrate, risk stratification, or ablation in repaired tetralogy of Fallot. Studies on atrial arrhythmias, non-rTOF congenital populations, and pediatric populations < 12 years of age were excluded unless they specifically discussed substrate biology or contemporary surgical practice. Synthesis is presented as a narrative integration of the included literature, with a comparative table of the reactive and proactive strategies, a future-directions matrix, and a clinical decision framework.

## 3. The Arrhythmogenic Substrate in rTOF

### 3.1. Anatomical Isthmuses: The Four Critical Corridors

**Figure 1:**
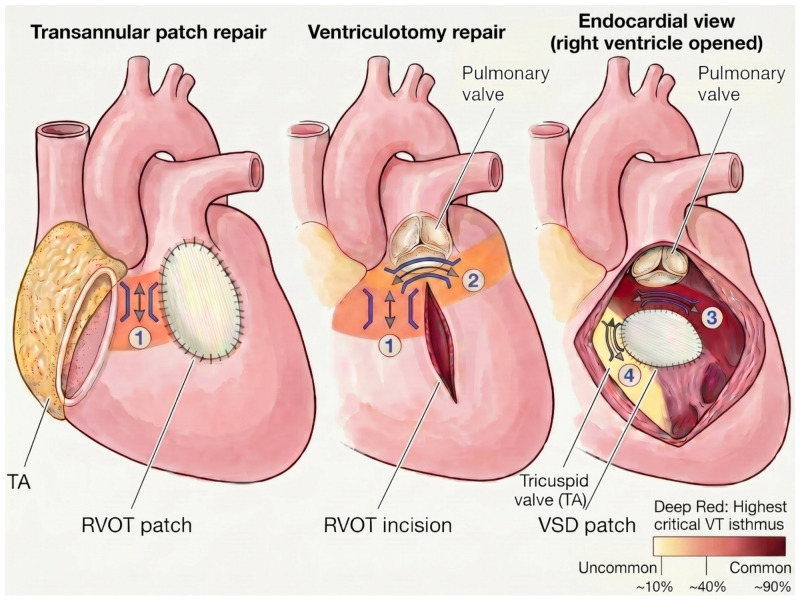
Schematic representation of the four anatomical isthmuses (AI) underlying ventricular tachycardia in repaired tetralogy of Fallot, shown in relation to two common surgical repair strategies. (**Left**) Transannular patch repair, in which the ventriculotomy and pulmonary annulus are bridged by a non-conducting patch, eliminating the corridor between the RVOT incision and the pulmonary valve and leaving isthmus 1 (between the RVOT patch and the tricuspid annulus) as an AI. (**Middle**) Limited ventriculotomy repair with preservation of the pulmonary annulus, in which both isthmus 1 (RVOT incision–tricuspid annulus) and isthmus 2 (RVOT incision–pulmonary annulus) can be present. (**Right**) Endocardial view of the opened right ventricle illustrating Isthmus 3 (VSD patch–pulmonary annulus) and isthmus 4 (VSD patch–tricuspid annulus). The color gradient encodes the reported frequency with which each AI harbors the critical isthmus of clinical or inducible VT, with deep red indicating the highest frequency (~90%) and pale yellow indicating uncommon involvement (~10%); Isthmus 3 is the most consistently arrhythmogenic substrate, identified in approximately 94% of postmortem rTOF specimens and implicated as the critical circuit in 80–90% of inducible VTs, whereas isthmus 1 (~88%), isthmus 2 (~25%) and isthmus 4 (~13%) are comparatively uncommon. AI, anatomical isthmus; RVOT, right ventricular outflow tract; TA, tricuspid annulus; VSD, ventricular septal defect; VT, ventricular tachycardia.

VT in rTOF is predominantly due to reentry. The circuits responsible for reentrant VTs involve anatomical isthmuses (AI) that are created from electrical barriers that are anatomical (tricuspid and pulmonary valve annuli) or due to surgical scars or patches (ventriculotomy, VSD patch, RVOT patch) [11,14]. The four AI that have been distinctly described in rTOF are as follows: (1) Isthmus 1: between the transannular/ventriculotomy scar and tricuspid annulus, (2) Isthmus 2: between the RVOT incision (or patch) and pulmonary annulus, (3) Isthmus 3: between the VSD patch (or septal scar) and pulmonary annulus, and (4) Isthmus 4: between the VSD patch and tricuspid annulus [8,11] (Figure 1).

Isthmus 3 is the most consistently identified, present in approximately 94% of postmortem rTOF specimens [14] and is implicated as the critical circuit isthmus in 80–90% of inducible VTs in systematic mapping series [16,17]. Isthmus 1 is present in 88% of cases [14]. However, isthmus 2 is comparatively uncommon, accounting for approximately 25% of cases [14] and reflecting contemporary surgical practice in which the ventriculotomy typically extends across the pulmonary annulus as a transannular incision or patch, thereby eliminating the intervening corridor of viable myocardium between the ventriculotomy edge and the pulmonary annulus. Isthmus 4 is the least common accounting for nearly 13% of patients [14] as the rim of myocardium between the VSD patch and the tricuspid annulus is frequently absent or vestigial in the rTOF, given the proximity of the malalignment VSD to the septal leaflet of the tricuspid valve.

### 3.2. Slowly Conducting Anatomical Isthmuses

Not every anatomical isthmus is arrhythmogenic. The feature that separates an inert AI from one capable of supporting reentrant VT is slow conduction. SCAIs are defined as anatomic isthmuses with a local conduction velocity (CV) below 0.5 m/s, and these are the dominant electrophysiological substrate for monomorphic VT in rTOF [14].

The relationship between SCAI and clinical VT is robust and reproducible. In an early cohort (n = 74), all mapped VT circuits traversed an isthmus with conduction velocity < 0.5 m/s, whereas nearly all isthmuses in patients without inducible VT had conduction velocity > 0.5 m/s [8]. SCAIs co-localized with the critical isthmus of induced VT in 90% of cases [8]. Over 4-year follow-up, patients without a SCAI were free of VT, while approximately half of those with at least one SCAI developed VT [8]. Similar to those findings, in a prospective multicenter study consisting of pre-PVR cohorts (n = 120), sustained VT was inducible in 22.5% of patients, among whom SCAI3 was present in 90% [17].

There are two practical implications of these observations: First, the absence of a SCAI on detailed EAM carries a high negative predictive value (NPV) for VT inducibility and subsequent clinical events [4], supporting a role for substrate mapping in risk stratification of asymptomatic patients. Second, because the substrate is anatomically discrete and accessible, SCAI is a viable ablation target, as transecting the isthmus interrupts the reentrant loop and can eliminate the substrate before VT manifests clinically.

### 3.3. Age-Related Substrate Evolution

The arrhythmogenic substrate in rTOF evolves progressively throughout life. Conduction slowing and isthmuses transition from electrically inert corridors to clinically arrhythmogenic SCAI occur in parallel with ongoing myocardial fibrosis and right ventricular remodeling [6,7,8]. Younger patients have less fibrotic scars, wider isthmuses, and SCAIs may not yet be fully formed. In a cohort of children and young adults with rTOF (n = 55, median age of 16 years), only 29% had an identifiable SCAI and monomorphic VT was inducible in just 8 patients (15%) [18]. However, this profile could shift as age increases. In a multicenter pre-PVR cohort (n = 180, mean age 39 ± 14 years), VT isthmus conduction velocity declined by 0.08 m/s per decade (*p* = 0.008), VT cycle length increased by 15 ms per decade (*p* = 0.005), and the prevalence of inducible VT rose progressively across decades of life (*p* < 0.001) [6]. At least one SCAI was reliably identifiable beyond age 32, and inducibility was best discoverable at an age threshold of 42 years with sensitivity of 68%, and specificity of 66% [6]. Therefore, the appearance of a SCAI on EAM precedes VT inducibility by several years, providing a window during which the substrate is established and mappable but has not yet caused sustained reentry [6].

**Figure 2:**
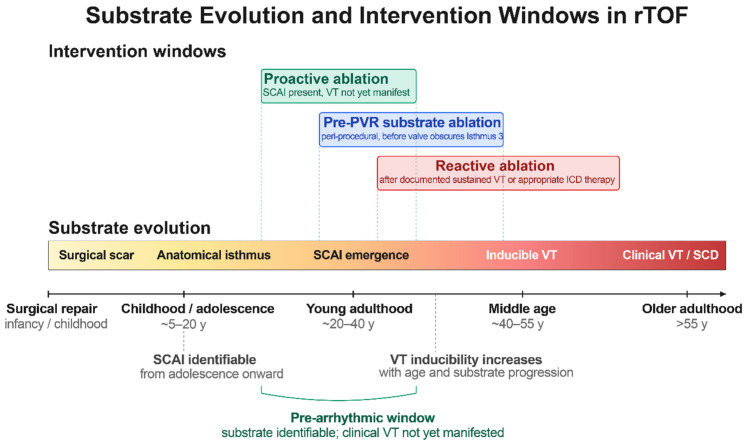
Conceptual schematic of arrhythmogenic substrate evolution and the corresponding windows for ablative intervention across the lifespan in repaired tetralogy of Fallot. The lower band depicts progressive maturation of the substrate from inert surgical scar in infancy and childhood, through formation of anatomical isthmuses and emergence of slowly conducting anatomical isthmuses (SCAI) during adolescence and adulthood, to inducible and ultimately clinically manifest VT or sudden cardiac death in middle and older age. SCAI become identifiable on electroanatomical mapping from adolescence onward, and the prevalence of inducible VT increases with age in parallel with ongoing fibrosis and right ventricular remodeling. The two intervention strategies are aligned to this trajectory. Proactive ablation (green) targets the SCAI before any clinical arrhythmia, typically electively in young adulthood or when the substrate is first demonstrated. Specifically Pre-PVR proactive substrate ablation (blue) is performed peri-procedurally at the time of pulmonary valve replacement, before transcatheter or surgical valve deployment may render Isthmus 3 anatomically inaccessible. Reactive ablation (red) follows documented sustained VT, VT storm, or appropriate ICD therapy, and is feasible at any point after clinical VT manifests. The age ranges shown are intended as an illustrative depiction of typical disease trajectory in contemporary cohorts and should not be interpreted as discrete thresholds; individual timing varies with era of repair, surgical technique, residual hemodynamic lesions, and genetic background. ICD, implantable cardioverter-defibrillator; PVR, pulmonary valve replacement; rTOF, repaired tetralogy of Fallot; SCAI, slowly conducting anatomical isthmus; SCD, sudden cardiac death; VT, ventricular tachycardia.

This temporal pattern has direct clinical implications. Mapping performed too early may underestimate latent substrate that will emerge with continued remodeling; conversely, deferring evaluation until VT becomes inducible misses the interval during which substrate elimination is most likely to alter the natural history. Targeting an established but not-yet-inducible SCAI offers a substrate that is both anatomically tractable and temporally pre-arrhythmic, providing a rationale for proactive evaluation in adults approaching middle age, particularly those with surgical risk factors or those scheduled for PVR (Figure 2).

### 3.4. Polymorphic VT: A Distinct Entity?

Polymorphic ventricular tachycardia (PMVT) and ventricular fibrillation (VF) represent a clinically important but mechanistically distinct subset of ventricular arrhythmias in rTOF. PMVT/VF accounts for approximately 15–20% of appropriately treated arrhythmias in ICD cohorts [13] with an actuarial event rate of 3.6% per year [19]. PMVT/VF can be inducible during programmed ventricular stimulation in 4–10% of patients [4,17].

The clinical interpretation of inducible PMVT/VF remains to be elucidated, as there is conflicting evidence in the literature. Earlier studies suggested that inducible PMVT/VF conferred a higher relative risk for clinical VT or SCD compared to monomorphic VT (RR 13 vs. 5) [4], supporting the view that PMVT/VF reflects diffuse, non-isthmus-dependent substrate. Contemporary data challenge this interpretation: they suggest that inducible PMVT/VF is not a single entity and that at least a subset is isthmus-mediated rather than reflecting diffuse non-isthmus substrate. In a multicenter pre-PVR cohort (n = 186, sustained PMVT inducible in 15%), patients with inducible PMVT actually exhibited a less advanced substrate compared to those with inducible VT, characterized by more contemporary surgical era (*p* = 0.008), absence of palliative shunts (*p* = 0.01), lower RV/LV end-diastolic volume ratio (*p* = 0.02), and faster anatomical isthmus conduction velocity (*p* = 0.03) [13]. Furthermore, transiently organized PMVT, defined as ≥ 3 consecutive beats of similar morphology at episode onset, was significantly more frequent in patients harboring at least one anatomical isthmus (median 3 vs. 0 beats, *p* = 0.001) and diminished after catheter ablation (58% at baseline vs. 8% at follow-up EPS, *p* = 0.001), suggesting that a subset of inducible PMVT may be initiated by an isthmus-mediated trigger that secondarily degenerates into a polymorphic morphology [13].

For the purposes of this review, PMVT/VF is acknowledged as a distinct component of the rTOF arrhythmic substrate spectrum and is not central to the proactive-versus-reactive isthmus ablation framework. Its presence at EPS, however, should prompt careful and individualized ICD decision making. The induction of PMVT/VF should also prompt caution against assuming that SCAI ablation alone fully mitigates arrhythmic risk and should strengthen individualized consideration of ICD protection, because PMVT/VF may fall outside the standard isthmus-ablation paradigm [20].

## 4. Risk Stratification: Selecting Candidates for Intervention

### 4.1. Clinical Risk Scores

Selecting patients who are likely to benefit from a proactive ablation strategy depends on accurate identification of those at arrhythmic risk. Current risk-stratification tools were developed primarily to inform ICD decision-making rather than ablation candidacy, and their performance in this latter role is currently under active investigation.

Established risk factors for VT/SCD in rTOF include QRS duration > 180 ms (a surrogate for RV enlargement and fibrosis), fragmented QRS, right or left ventricular systolic dysfunction on echo and history of syncope, non-sustained VT, or inducible sustained VT at programmed ventricular stimulation [7,9,19,21]. Quantification of RV or LV myocardial fibrosis using 3-dimensional high-resolution late gadolinium enhancement Cardiac Magnetic Resonance (LGE-CMR) also independently predict inducible VT and arrhythmic events [22].

Several composite scores integrate these variables: Khairy’s 12 point-score includes RV/LV dysfunction, non-sustained VT (NSVT), QRS > 180 ms, and inducible VT [19]. The PREVENTION-ACHD score is built from clinical and electrophysiologic variables (coronary artery disease, NYHA II/III heart failure, supraventricular tachycardia, systemic and subpulmonary EF < 40%, QRS ≥ 120 ms, QT dispersion ≥ 70 ms), albeit without CMR input [23]. The Ghonim survival model adds CMR-derived parameters including LGE burden [24]. Contemporary guidelines (AHA/ACC 2018, ESC 2020) recommend ICD implantation when defined thresholds are met (e.g., LVEF < 35%, symptomatic ventricular arrhythmias, inducible sustained VT), with LGE-CMR fibrosis acknowledged as an emerging modifier [9,25]. Risk scores however are heterogeneous and imperfect for identifying patients at high risk of arrhythmic events in rTOF [26]. In the present era, use of multiple criteria alongside adjunctive stratification strategies and multidisciplinary discussion is therefore critical.

Given that rTOF substrate evolves over decades, a single cross-sectional risk estimate is expected to underestimate the cumulative lifetime risk. A recent meta-analysis of 15 cohort studies (n = 1459) identified the presence of SCAI as a predictor of VT occurrence, with SCAI-based ablation markedly reducing occurrence (RR 0.11; 95% CI 0.03–0.33) [27]. EAM-based stratification has emerged as an individualized risk-stratification approach, in patients without prior clinical VT [8]. In a single-center cohort of rTOF patients without prior VT (n = 97), EAM and proactive SCAI ablation were performed and compared retrospectively against four contemporary risk-stratification strategies [28]. Application of the Khairy clinical score [19], AHA 2018 guidelines without LGE, AHA 2018 with LGE [9], and ESC 2022 [25] guidelines would have qualified 51%, 25%, 32%, and 49% of patients for ICD implantation, respectively. After EAM with SCAI transection where indicated, only 11% remained ICD candidates, and all four VT events during a median 58-month follow-up occurred in this residual high-risk group [28]. Their study was a retrospective single-center, non-randomized study, making it vulnerable to selection bias, and center-specific procedural expertise. However, their findings suggest that EAM-based substrate characterization could refine patient selection beyond what conventional risk scores can achieve and represents a basis for offering proactive ablation [28].

No published risk score has been prospectively validated specifically for selecting candidates for prophylactic ablation. In current practice, the decision rests on a composite assessment that combines conventional clinical and imaging risk factors with EAM findings when available, applied within a shared decision-making framework. Whether a dedicated, prospectively derived score for ablation candidacy will outperform this hybrid approach is an important target for future investigation.

In selected patients with repaired tetralogy of Fallot who remain in a phase of uncertain or transient arrhythmic risk, for example after substrate evaluation, after PVR, after incomplete substrate elimination, or while awaiting repeat EPS, the wearable cardioverter-defibrillator (WCD) may provide temporary protection against SCD while avoiding premature ICD implantation. Recent evidence supports WCD therapy in patients with transiently increased arrhythmic risk and highlights the value of multiparametric reassessment to avoid unnecessary permanent defibrillator implantation [29,30].

Importantly, ablation and ICD therapy should not be considered interchangeable. ICDs do not eliminate the arrhythmogenic substrate, but they protect against fatal ventricular arrhythmias, including polymorphic VT/VF or late events that may not be fully addressed by isthmus ablation. Conversely, successful SCAI ablation may reduce VT burden and refine ICD candidacy, but it should not automatically replace ICD therapy in patients with ventricular dysfunction, extensive fibrosis, inducible PMVT/VF, incomplete bidirectional block, or residual substrate. Decision on deferring ICD implantation after a successful ablation should be done on a case-by-case basis with appropriate shared decision making [31].

### 4.2. Role of Programmed Ventricular Stimulation

Programmed ventricular stimulation (PVS) remains a central component of risk assessment in rTOF, particularly when non-invasive markers or risk scores yield intermediate risk or conflicting results. In a multi-center cohort of 252 patients undergoing PVS, inducible sustained VT had a NPV of 91.5% and positive predictive value (PPV) of 55.2% for future VT/SCD [4]. VT Inducibility conferred a five-time higher risk for adverse events, and lower event-free survival at 15 years (50%) compared to non-inducibility (89%) [4]. The high NPV makes a negative study reassuring, while the modest PPV reflects the heterogeneity of patients in whom inducibility is observed. Inducible patients are typically directed toward ICD implantation, ablation, or both, and PVS therefore plays a dual role in risk stratification and procedural planning. Moreover, PVS is most informative when integrated with clinical risk factors and imaging rather than applied in isolation, and its results inform, but do not solely determine, decisions regarding ICD implantation and ablation candidacy.

### 4.3. Cardiac MRI–Based Substrate Identification

LGE-CMR is an emerging non-invasive modality for substrate characterization in rTOF, allowing direct quantification of the right ventricular scar burden that underlies reentrant VT. Ghonim et al. reported that RV LGE volume > 20 cm^3^ predicted VT inducibility with 72% sensitivity and 81% specificity [22]. More recently, 3D LGE-CMR techniques predict the presence of SCAIs non-invasively with 100% sensitivity and 90% specificity [32]. Pre-procedural CMR planning could spare approximately 70% of patients an EPS that would yield no substrate [32]. CMR-derived maps of RV anatomy and fibrosis can be co-registered with EAM to guide ablation [32,33]. Despite these promising data, several limitations remain relevant to clinical implementation, including the suboptimal image quality in patients with prior cardiac devices or arrhythmia, variable inter-center reproducibility of fibrosis quantification, and constrained access in lower-resource settings.

In resource-limited settings, where 3D LGE-CMR and high-density EAM, are not available, simple clinical, historical and ECG/ECHO based metrics can be used to risk stratify patients and decide who merits advanced evaluation. Although specific guidance e on a non-invasive/non-CMR threshold that merits escalation of risk stratification techniques, a pragmatic pathway relies on careful serial clinical assessment, 12-lead ECG QRS duration and fragmentation, ambulatory rhythm monitoring, echocardiographic assessment of ventricular size and function, conventional CMR when available, and early referral to regional ACHD-electrophysiology centers for patients with prior VT, NSVT, syncope, progressive ventricular dysfunction, marked QRS prolongation, or planned native-RVOT PVR.

## 5. Reactive VT Ablation: Evidence and Outcomes

### 5.1. Catheter-Based VT Mapping and Ablation Techniques

VT ablation in rTOF is usually performed via right femoral venous access for endocardial mapping of the right ventricular outflow tract and surrounding scar. Retrograde aortic or transseptal access to the left ventricle is occasionally required, either when the substrate extends to the LV side of the conal/outlet septum, or when right-sided ablation fails to achieve transmural lesions across the thickened septum adjacent to the VSD patch, particularly along Isthmus 3, where additional energy delivery from the LV endocardium can complete the lesion set [10]. The procedure workflow typically consists of substrate identification, isthmus characterization, VT induction and activation/entrainment mapping when tolerated, followed by ablation with confirmation of conduction block.

Substrate identification is performed by three-dimensional EAM during sinus or paced rhythm. Bipolar voltage maps delineate dense scar (typically < 0.5 mV) and the surgical and physiological barriers (transannular incision or RVOT patch, VSD patch, tricuspid and pulmonary annuli) that bound the four anatomical isthmuses. Non-capture during pacing at 10 mA at 2 msec can also confirm unexcitable tissue and patches [11]. Once the isthmuses are anatomically defined, each is interrogated for its CV by dividing the distance between two sites on the opposing site of the isthmus by the difference of the local activation time of these sites. CV assessment serves both as a substrate-characterization tool and as the principal determinant of which isthmuses warrant ablation [8]. Mapping resolution has been substantially augmented by ultra-high-density multipolar catheters (e.g., HD Grid, Octarray, Optrell, Orion), which allow rapid acquisition of thousands of points and improved detection of narrow conduction channels and late potentials within the rTOF substrate [17]. Pre-procedural integration of LGE-CMR with the electroanatomic shell allows direct co-registration of imaged fibrosis with electrical substrate. In a validated rTOF cohort, three-dimensional LGE-CMR identified 95% of anatomical isthmuses at a 60% maximum-signal-intensity cutoff, supporting its role in pre-procedural planning [34].

If sustained VT can be induced and is hemodynamically tolerated, conventional activation and entrainment mapping confirm the critical isthmus. When VT is non-tolerated or non-inducible, substrate-based ablation targets the identified SCAI. Ablation is delivered as a linear lesion set transecting the isthmus from one electrical barrier to the other. The procedural endpoints are bidirectional conduction block across all targeted isthmuses, confirmed by differential pacing and remapping, and non-inducibility on repeat programmed stimulation. Despite advances in technology, the procedure remains technically demanding due to complex RV geometry, variability of surgical repair, and proximity of critical structures (such as coronary arteries and the conduction system), and is best performed in centers with combined ACHD and adult electrophysiology expertise.

### 5.2. Clinical Outcomes of Reactive Catheter Ablation

Reactive VT ablation in experienced centers achieves consistently high acute procedural success and durable but imperfect long-term arrhythmia control. Early series demonstrated that all induced VTs traversed an anatomical isthmus and that linear ablation transecting these isthmuses abolished all VTs, with 91% VT-free survival over a mean follow-up of 30 months (n = 11) [11]. Long-term outcomes from a single-center cohort spanning two decades reported acute success in 82% of patients (n = 34), with freedom from death and arrhythmia recurrence of 94%, 81%, and 70% at 5, 10, and 20 years, respectively, and overall survival of 91% at 20 years [12]. A pooled analysis of 15 cohort studies (n = 1459) confirmed that SCAI-based catheter ablation substantially reduces VT recurrence compared with non-substrate-targeted approaches (RR 0.11; 95% CI 0.03–0.33), without statistically significant evidence for reduced SCD or all-cause mortality [27].

Procedural safety has been favorable across reported series. Major complication rates are 5–10% in pooled data. These rates are consistent with contemporary studies on VT ablation in non-congenital patient populations [35]. These complications consist predominantly of vascular access events; cardiac perforation and complete heart block are uncommon, the latter in part because most patients already carry RBBB and ablation lesions are oriented away from the conduction system [27,36]. No procedure-related deaths have been reported [11,12].

Despite favorable acute success, reactive VT ablation does not eliminate long-term arrhythmic risk, and recurrent ventricular events may occur. In the Laredo cohort, two sudden cardiac deaths and four VT recurrences were observed over a mean follow-up of 9.5 years [12]. Recurrence of VT occurs due to (1) ongoing substrate evolution: new SCAIs may emerge with progressive remodeling, fibrosis, and surgical scar maturation, (2) patients with extensive baseline substrate or ventricular dysfunction remaining at residual risk even after successful ablation and (3) incomplete ablation during the index procedure with re-connection of the ablation line. Baseline LVEF < 60% was the strongest independent predictor of arrhythmia recurrence (HR 16.4; 95% CI 1.8–147; *p* = 0.01) [12,27,37]. Accordingly, post-ablation surveillance, redo mapping when clinically indicated, and ICD implantation for secondary prevention remain integral to long-term management.

**Figure 3:**
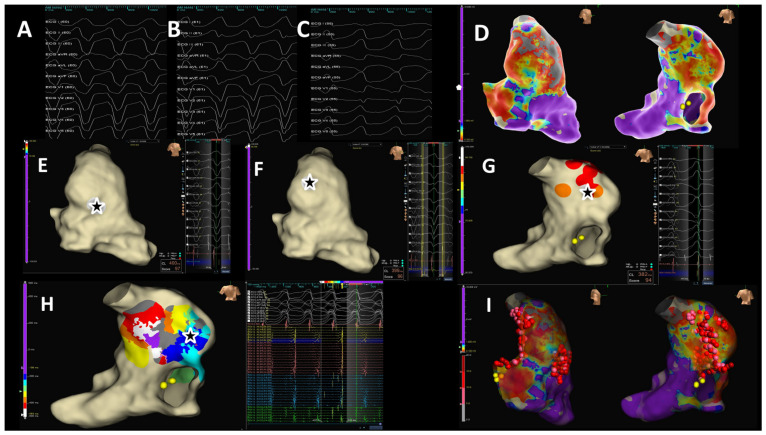
Example of a reactive VT ablation. The patient is a 65-year-old man with rTOF (repaired at age 9) presenting with sustained VT, who underwent secondary-prevention ICD implantation at another institution prior to arrival to our service. Cardiac magnetic resonance showed a dilated right ventricle with transmural late gadolinium enhancement of the RVOT and septum; LVEF was 68%. Three monomorphic VT morphologies were inducible during the EP study: VT1 that had similar morphology with the clinical VT; VT2 and VT3 were also induced on programmed ventricular stimulation. (**A**–**C**) Twelve-lead ECG of VT1 (clinical), VT2, and VT3, each with a distinct QRS morphology. (**D**) Right ventricular bipolar voltage map (0.1–1.5 mV) demonstrating extensive low-voltage scar involving the anterior RV wall and anatomical Isthmus 3, with preserved voltage inferiorly and basally. (**E**) Pace-mapping at the anterior RVOT, within anatomical isthmus 1, yielded a 97% match to VT. (**F**) Pace-mapping superior to this site, within the same anatomical isthmus 1, yielded a 96% match to VT2. (**G**) Pace-mapping at the lateral aspect of anatomical Isthmus 3 yielded a 94% match to VT3. (**H**) Limited activation map of VT3 with mid-diastolic potentials on the multipolar catheter, consistent with macroreentry within anatomical Isthmus 3. Together, panels (**E**–**H**) localize VT1 and VT2 to anatomical isthmus 1 and VT3 to anatomical Isthmus 3, two adjacent corridors visible in panel (**D**). (**I**) Final ablation set: linear lesion transecting anatomical Isthmus 3 and homogenization of anatomical isthmus 1. Non-inducibility and bidirectional block across anatomical Isthmus 3 were achieved. The patient remained free of VT and ICD therapies at 6-month follow-up. Yellow spheres mark the His bundle; stars in panels (**E**–**G**) denote the pace-mapping reference sites and in (**H**) the site where the mid-diastolic potentials were recorded. CMR, cardiac magnetic resonance; ECG, electrocardiogram; ICD, implantable cardioverter-defibrillator; LGE, late gadolinium enhancement; LVEF, left ventricular ejection fraction; rTOF, repaired tetralogy of Fallot; RV, right ventricle; RVOT, right ventricular outflow tract; VT, ventricular tachycardia.

Figure 3 depicts a representative case of reactive VT ablation.

## 6. Proactive (Prophylactic) VT Ablation: Emerging Evidence

### 6.1. Rationale

The rationale for proactive ablation has been developed based on three converging observations. First, the arrhythmogenic substrate, defined by the presence of one or more SCAIs, can be identified by EAM-guided programmed ventricular stimulation in asymptomatic patients well before the first clinical VT episode [6,8]. Second, the substrate burden in patients without prior clinical VT is lower than in those who have already manifested arrhythmia, with fewer and shorter SCAIs, which translates into a technically simpler ablation and a higher likelihood of achieving complete bidirectional block across all targeted isthmuses [38]. Third, anatomical changes after PVR, particularly transcatheter valves deployed across the native right ventricular outflow tract, can render SCAI3 inaccessible to subsequent endocardial catheter ablation, effectively closing the therapeutic window for substrate modification once the valve is in place [7]. Reflecting these considerations, the published experience with proactive ablation in rTOF has accumulated almost entirely in the pre-PVR setting, with substrate mapping and ablation timed at, or immediately before, surgical or transcatheter valve replacement; data on stand-alone elective proactive ablation in asymptomatic high-risk patients without a planned PVR remain limited.

**Figure 4:**
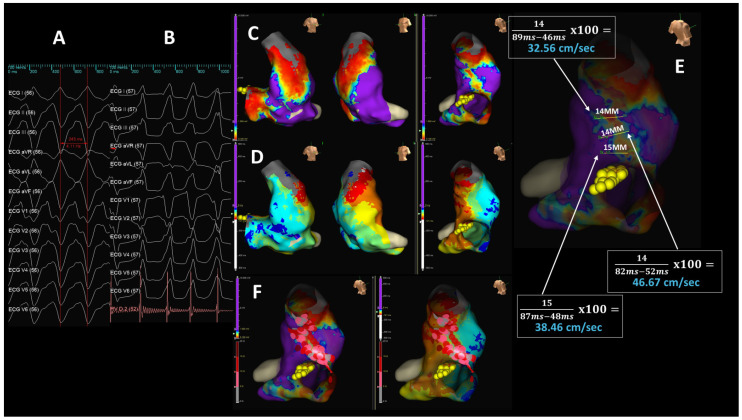
Example of a proactive VT ablation. Proactive EPS and substrate-based VT ablation in a 47-year-old woman with rTOF and no prior clinical VT performed prior to pulmonary valve replacement. Two distinct monomorphic VT morphologies were inducible during programmed ventricular stimulation. (**A**) Twelve-lead ECG of VT1 (cycle length 243 ms), with left bundle branch block morphology and superior axis. (**B**) Twelve-lead ECG of VT2, non-sustained, with a distinct morphology of left bundle branch block and inferior axis consistent with a separate exit site. (**C**) Right ventricular bipolar voltage map (0.1–1.5 mV scale) demonstrating a heterogeneous low-voltage area at the region of AI3 as well as a large area of dense scar along the anterior RV most likely corresponding to an RVOT patch. (**D**) Right ventricular isochronal late activation map (ILAM) demonstrates marked isochronal crowding within the region of AI3, indicating slow conduction. (**E**) Local conduction velocity measurements across the AI3, calculated as the distance between paired sampling points divided by the difference in local activation time. All three measurements yielded conduction velocities below 0.5 m/s, establishing the presence of SCAI3 and identifying it as the dominant arrhythmogenic substrate. (**F**) Final ablation lesion set, with linear radiofrequency lesions transecting SCAI3. Procedural endpoints of bidirectional block, confirmed by differential pacing, and non-inducibility on repeat programmed stimulation were both achieved. The patient subsequently underwent uncomplicated pulmonary valve replacement. Yellow spheres mark the anatomical location of the His bundle. ECG, electrocardiogram; ILAM, isochronal late activation map; PVR, pulmonary valve replacement; rTOF, repaired tetralogy of Fallot; RV, right ventricle; RVOT, right ventricular outflow tract; SCAI, slowly conducting anatomical isthmus; VT, ventricular tachycardia.

The conceptual precedent for performing ablation before a planned cardiac procedure is well established in congenital electrophysiology. In Ebstein’s anomaly, accessory-pathway ablation is routinely performed before surgical cone repair because subsequent anatomical reconstruction obscures pathway localization and complicates redo mapping [39]. The pre-PVR proactive ablation strategy in rTOF applies the same logic: a defined arrhythmogenic substrate, accessible at the time of an unrelated planned intervention, is best addressed before the procedure that will alter or obscure it. By transecting potential reentry corridors before they become clinically relevant, proactive ablation aims to prevent the first VT event. This is supported by a single-center cohort of rTOF patients without prior VT (n = 97), in which proactive EAM and SCAI ablation reduced eligibility for primary prevention ICD implantation from 25 to 51% (depending on the guideline applied) to 11% [28]. Figure 4 depicts a representative case of proactive VT ablation.

### 6.2. Systematic Pre-PVR Electrophysiology Studies and Proactive Ablation

The clinical utility of systematic pre-PVR EPS and preventive substrate ablation has been examined in prospective cohorts [17,36,40], providing convergent evidence on inducibility yield, anatomical substrate localization, and post-ablation outcomes. In a multicenter cohort of 120 adults referred for PVR, programmed ventricular stimulation induced sustained VT in 22.5% of patients; SCAI3 was present in 90% of cases. History of atrial arrhythmia (OR 8.56; 95% CI 2.43–34.73) and pulmonary annulus diameter > 26 mm (OR 5.05; 95% CI 1.47–21.69) emerged as independent predictors of inducibility. The presence of SCAI altered clinical management in 19.2% of patients, leading to catheter ablation in 15.0%, surgical cryoablation in 2.5%, and primary-prevention ICD implantation in 7.5%. Repeat EPS approximately five months after PVR was non-inducible in 88.9% of the retested patients, and no sustained ventricular arrhythmia occurred during a median follow-up of 13 months [17]. A separate single-center cohort of 77 pre-PVR patients followed for a median of 74 months. Sustained VT was inducible in 23.4% of patients, and ablation was performed in 28 patients overall, including selected non-inducible patients with slow conduction (catheter ablation (n = 5), surgical cryoablation (n = 9), or both (n = 14)). Over 74 ± 40 months, no SCD occurred, 3 sustained VAs occurred, all in patients inducible at initial EPS, and none occurred in the non-inducible group [36]. However, in another pre-PVR cohort from two centers (n = 70) who underwent pre-PVR EPS, 34 (49%) had inducible sustained VT: 25 monomorphic VT and 9 PMVT [40]. If inducible, patients underwent empirical surgical cryoablation during PVR, followed by post-PVR EPS to guide ICD. Cryoablation was performed in 31 patients, and post-PVR EPS was positive for inducible VT in 14 patients, all of whom then underwent ICD implantation. During 6 years follow-up, 3 of 14 patients (21%) who received ICDs had appropriate ICD shocks for symptomatic VT [40]. High rate of inducibility after cryoablation in Sandhu et, al study [40] suggests that empirical surgical lesions may be insufficient, while Bouyer [36], et al. and Waldmann, et al. [17] suggest EAM-guided targeted ablation with confirmation of block may improve outcomes. Whether some patients harbor substrates too extensive or anatomically complex to be eliminated by a single procedure, such as extensive transannular scar, multiple SCAIs, or substrate extending beyond the four canonical isthmuses, remains to be determined as current series are underpowered to resolve.

Despite differences in design and follow-up, cohorts converge on similar findings. First, 20–50% of rTOF patients arriving for PVR harbor an arrhythmogenic substrate sufficient to induce sustained VT [17,36,40], even without prior clinical arrhythmia. This, in the authors’ opinion, justifies consideration of a systematic rather than purely selective approach to EPS in this population. Second, the predominance of Isthmus 3 as the critical reentry corridor concentrates the technical target and aligns with the anatomical concern that PVR will subsequently may render this isthmus inaccessible. Third, the combination of EPS findings with readily available clinical and imaging variables, particularly atrial arrhythmia history and pulmonary annulus dimension, may permit pre-test risk stratification and guide which patients warrant the most thorough mapping protocol. Finally, the durability of negative outcomes among non-inducible patients out to six years suggests that a comprehensive pre-PVR EPS, when negative, identifies a low-risk subgroup in whom ICD implantation may not be required, an argument also supported by contemporary EAM-based management cohorts [28].

Limitations of the current evidence are: first, the studies are observational, with the larger cohort having a relatively short follow-up and the longer-follow-up cohort being either single-center or small cohort; second, neither directly compares an EPS-guided proactive strategy against standard care in a randomized fashion, and the heterogeneity of ablation modality, catheter, surgical, or both, reflects pragmatic decision-making rather than a uniform protocol. A randomized comparison of pre-PVR EPS and substrate ablation against guideline-directed care, with hard arrhythmic endpoints and pre-specified ICD criteria, remains an important unmet need.

### 6.3. Proactive SCAI Ablation Outside of the Pre-PVR Setting

Beyond the pre-PVR setting, dedicated evidence for proactive ablation is sparse. The published cohorts that support proactive VT ablation in rTOF are derived from mixed populations in which pre-PVR patients predominate. In these cohorts non-PVR cases form a minority subset that has not been separately analyzed for outcomes. No prospective study has been designed specifically to evaluate proactive ablation in patients without a planned valve intervention.

Limited data suggest, but do not establish, that the favorable outcomes observed in pre-PVR proactive cohorts may extend to non-PVR settings. In the largest controlled comparison to date, of 57 patients who underwent proactive EAM and SCAI ablation, 45 (79%) were referred before planned PVR, while 12 (21%) had non-PVR indications: five for clinical risk factors alone and seven for concomitant atrial arrhythmia ablation or pacemaker implantation. Across the entire prophylactic cohort, no patient experienced the composite arrhythmic endpoint over a median 21-month follow-up. This favored the prophylactic strategy over a historical PVS-only approach [38]. Notably, four of ten composite endpoints in the historical comparator group occurred within the first 14 months of follow-up, paralleling the entire follow-up window of the prophylactic cohort and arguing that the outcome difference reflects a strategy effect rather than an artifact of unequal observation time. A separate single-center cohort of 97 rTOF adults without prior VT included approximately one-third of patients undergoing PVR during the study window, with the remainder evaluated for substrate identification under standard institutional protocol; outcomes were favorable across the cohort, but the report does not stratify Kaplan–Meier outcomes by PVR status [28]. Among 55 children and young adults under 30 years of age, 10 (18%) were referred for risk stratification independent of PVR planning, and SCAI 3 was identifiable even at this age, with two VT recurrences across the entire cohort over a median 5.3-year follow-up [18].

Several special patient populations merit consideration for proactive VT ablation but have not been formally studied. The first is the asymptomatic patient with high-risk imaging or electrocardiographic features, including extensive LGE on CMR, QRS duration > 180 ms, fragmented QRS, or non-sustained VT, in whom no PVR is planned. The second is a patient referred for atrial arrhythmia ablation, in whom right ventricular EAM and ablation can be added to an already invasive procedure with limited additional risk; this scenario accounted for seven of the twelve non-PVR prophylactic ablations in the largest published series. The third is an adolescent or young adult in whom anatomic Isthmus 3 has been identified at an age when remodeling is ongoing, and the trajectory toward inducibility is not yet defined; the optimal timing of intervention in this group is unresolved. The fourth is a patient with prior unsuccessful or incomplete pre-PVR ablation, in whom remapping after substrate evolution may identify new targets. None of these scenarios have been the subject of a dedicated cohort with prespecified endpoints.

Several questions are central to defining the role of proactive ablation outside the pre-PVR context and remain unanswered. Which combination of clinical, imaging, and electrophysiological criteria should be used to select asymptomatic patients for elective EAM? At what age or stage of substrate evolution does the procedural risk-benefit balance favor intervention? Should proactive substrate evaluation be repeated longitudinally in patients with negative initial mapping, given evidence that anatomical isthmuses can transition to slow conduction with continued remodeling? Does proactive ablation in non-PVR contexts deliver the same magnitude of ICD-avoidance and event-free survival observed in pre-PVR cohorts, or does the absence of concurrent hemodynamic intervention attenuate the benefit? Answering these questions requires prospective multicenter studies with standardized selection criteria, uniform ablation endpoints (bidirectional block, non-inducibility), structured longitudinal follow-up, and prespecified comparison against guideline-directed risk stratification. Until such data are available, proactive ablation outside the pre-PVR setting should be considered an individualized strategy applied at experienced congenital electrophysiology centers, with shared decision-making and explicit acknowledgment of the evolving evidence base (Table 1).

### 6.4. Surgical Substrate Ablation Concomitant with Pulmonary Valve Replacement

Surgical ablation of arrhythmogenic substrate at the time of PVR represents a complementary proactive strategy, leveraging direct surgical exposure and the ability to deliver transmural lesions. Most reported series target SCAIs identified preoperatively by EAM-guided programmed ventricular stimulation, with surgical radiofrequency ablation or cryoablation performed concomitantly with PVR. In a single-center cohort of 20 rTOF patients with inducible VT and risk factors for ventricular arrhythmia who underwent PVR with concomitant intraoperative radiofrequency ablation of preoperatively mapped SCAIs, freedom from ventricular arrhythmia was 94% at 1 year and 89.5% at 5 years over a median 6.5-year follow-up. Postoperative EPS demonstrated persistent inducibility in 15.7% of patients who received an ICD [41]. A separate retrospective analysis of 205 rTOF patients undergoing PVR, 22 of whom received surgical RVOT cryoablation at the time of valve replacement, reported a single arrhythmic event in the cryoablation group over the follow-up period, leading the authors to conclude that surgical cryoablation does not increase arrhythmic events and may be protective in selected high-risk patients [42]. However, group sizes were small, the cryoablation cohort was selected on the basis of clinical risk factors rather than preoperative substrate mapping, and the comparison was not adjusted for these differences, limiting causal inference. Important caveats apply to surgical ablation as a strategy. Its use is limited to patients undergoing surgical, rather than transcatheter, PVR; cryoablation lesions that are anatomically guided and may be less precise than EAM-targeted catheter ablation; and the absence of randomized data leaves uncertainty about the incremental benefit over PVR alone in patients without preoperative inducibility. As transcatheter PVR becomes the dominant approach in eligible patients, the role of surgical ablation is likely to be reserved for those undergoing surgical PVR for anatomical or hemodynamic reasons rather than clinical risk factors alone.

### 6.5. CATAPULT-TOF: The Prospective Trial

The CATAPULT-TOF study (Catheter Ablation of Ventricular Tachycardia Before Transcatheter Pulmonary Valve Replacement in Repaired Tetralogy of Fallot) [7] is the first prospective multicenter study designed to evaluate a uniform, algorithmic strategy of preemptive substrate ablation before transcatheter pulmonary valve placement in the native right ventricular outflow tract. It is endorsed by the Pediatric and Congenital Electrophysiology Society and the International Society of Adult Congenital Heart Disease. The study plans to enroll 188 adults with rTOF undergoing planned native-RVOT PVR. All participants undergo pre-PVR EPS with EAM and direct measurement of left ventricular end-diastolic pressure; ablation is then directed by the substrate findings, with transection of the SCAI and/or VT critical isthmus when monomorphic VT or SCAI is identified, and ablation of all SCAIs when only polymorphic VT is inducible, before PVR deployment. Repeating EPS at 6 ± 3 months post-PVR reassesses inducibility and informs ICD decision-making. The primary outcome is the change in the Khairy 12-point SCD risk score [19] versus after the combined ablation-PVR strategy, with secondary aims addressing acute procedural safety, ablation efficacy, durability of bidirectional block, and the influence of surgical era on outcomes. The design hypothesizes that the strategy will achieve non-inducibility in approximately 80% of treated patients and meaningfully reduce the proportion meeting conventional criteria for primary-prevention ICD implantation. CATAPULT-TOF is positioned to provide the first rigorous prospective data on whether the protection observed with preemptive substrate ablation in single-center series translates into a reproducible, multicenter outcome benefit, and its results will be central to defining the role of proactive ablation in the contemporary peri-PVR setting.

## 7. Effect of Pulmonary Valve Replacement on Arrhythmic Burden

There is conflicting evidence on whether PVR reduces ventricular arrhythmia risk through reverse remodeling, reduced wall stress, and partial restoration of the hemodynamic milieu. The 2022 AHA Scientific Statement on arrhythmias in repaired tetralogy of Fallot concludes that PVR does not eliminate the slow conducting anatomical isthmus that underlies most monomorphic VT in this population and accordingly recommends substrate evaluation and ablation in conjunction with valve replacement [3]. The preponderance of matched and propensity-adjusted analyses argues against a meaningful effect of PVR on sustained VT in patients with established arrhythmogenic substrate. This is evident from a matched comparison of 98 patients who underwent late PVR for symptomatic pulmonary regurgitation and right ventricular dilation against 77 controls with similar age and baseline QRS duration. Over 272 patient-years of follow-up, the PVR group accumulated thirteen events (death or VT), and there was no significant difference between groups in VT (*p* = 0.32), death (*p* = 0.06, with the trend nearly favoring controls), or the combined endpoint (*p* = 0.21). QRS duration did not change after PVR, arguing that the electrical substrate for reentry was not meaningfully modified [43]. Subsequent international propensity-adjusted analysis of 977 patients showed similar results: the adjusted hazard ratio for the primary endpoint of death or sustained VT was 0.65 (95% CI 0.31–1.36; *p* = 0.25), and in patients not meeting consensus criteria for PVR, the secondary composite of heart failure and arrhythmia events was actually higher after valve replacement (HR 2.31; 95% CI 1.07–4.97; *p* = 0.03), raising the possibility that PVR performed outside guideline indications confers no benefit and may worsen non-arrhythmic outcomes [44]. A large, long-term national cohort of 1744 rTOF patients added an important nuance, finding that PVR reduced subsequent arrhythmic events only in patients without tachyarrhythmia or sudden cardiac arrest before valve replacement; in those with pre-PVR arrhythmias, the procedure conferred no antiarrhythmic benefit and overall arrhythmic risk remained higher in the PVR group than in the unoperated cohort, although survival was better once an arrhythmic event had occurred [45]. The same pattern, namely that established arrhythmogenic substrate predicts post-PVR events independent of valve replacement, was demonstrated in a single-center analysis of 205 patients undergoing PVR, in whom prior VT carried a hazard ratio of 4.7 (*p* = 0.004) for post-PVR arrhythmic events. A small subgroup of 22 patients receiving concomitant surgical RVOT cryoablation experienced only one event over the follow-up period, suggesting that the substrate, rather than the valve, is the operative target [42]. Reports that have shown apparent reduction in VT incidence after PVR have generally relied on protocols in which intraoperative cryoablation was performed in most or all patients with preexisting ventricular arrhythmia and have therefore conflated the antiarrhythmic effect of ablation with the hemodynamic effect of valve replacement [42].

Conversely, propensity-matched analysis of 1143 patients from the INDICATOR registry reported a substantial reduction in death or sustained VT after PVR (adjusted HR 0.41; 95% CI 0.21–0.81; *p* = 0.010), with the benefit concentrated in patients with advanced RV dilation (RV end-systolic volume index > 80 mL/m^2^) [46]. The French DAI-T4F ICD registry documented a 79% reduction in appropriate ICD therapy after PVR within an ICD-protected, predominantly secondary-prevention cohort [47]. These findings indicate that a hemodynamic effect on arrhythmia burden likely exists in selected subgroups, particularly when PVR is performed before extensive electrical remodeling has accumulated and substrate is still partially reversible. They do not, however, contradict the broader observation that once an arrhythmogenic substrate is established, valve replacement alone is insufficient to abolish ventricular arrhythmia.

The discrepancies among PVR studies likely reflect differences in surgical era, baseline substrate maturity, referral indication, valve modality, duration of follow-up, and patient selection. Cohorts enriched for advanced RV dilation may show benefit from relief of hemodynamic stress, whereas patients with established SCAI, prior sustained VT, extensive fibrosis, or older surgical scars may retain fixed reentrant corridors despite favorable reverse remodeling. These mechanisms explain why PVR may reduce arrhythmic burden in selected hemodynamic phenotypes without reliably eliminating monomorphic VT substrate.

Two implications follow for the proactive-versus-reactive ablation framework. The first is that PVR should not be expected to function as a stand-alone arrhythmia-prevention strategy in patients with identifiable substrate or prior VT, and the residual arrhythmic risk in these patients provides the strongest rationale for concomitant substrate ablation at the time of valve replacement. The second is that the apparent antiarrhythmic effect of PVR, when observed, is most evident in patients without prior arrhythmia and with substantial RV dilation, the same subgroup in which proactive substrate evaluation can identify a SCAI before clinical VT manifests, allowing both interventions to be combined within a single procedural plan. Whether PVR alone, performed earlier in the disease course in patients without identifiable substrate, can prevent the development of substrate over time, rather than only attenuating its consequences once formed, remains an open question that the existing observational literature is not designed to answer.

## 8. Proactive vs. Reactive: A Critical Comparison

Table 2 summarizes a comprehensive critical comparison of the two approaches. This should be interpreted not as a competition between two strategies for the same patient, but as a sequencing question that depends on where in the trajectory of arrhythmogenic substrate evolution the patient is encountered. Reactive ablation has a longer evidentiary track record, with two decades of consistent data showing high acute success and durable, though imperfect, long-term arrhythmia control. Its principal limitation is intrinsic to its trigger: by the time a patient presents with sustained VT, an ICD has typically been implanted, the substrate is mature, and a residual lifetime risk persists despite successful index ablation, particularly in patients with reduced ventricular function.

Proactive ablation addresses this temporal limitation by intervening before clinical VT manifests, while the substrate is anatomically tractable and procedurally accessible. The available evidence, although derived from observational single-center cohorts and one ongoing prospective trial, is internally consistent: SCAI transection in the absence of prior VT is technically feasible, demonstrably safe, and associated with high arrhythmia-free survival in patients with complete bidirectional block. The most clinically meaningful consequence reported to date is a substantial reduction in primary-prevention ICD candidacy, with all observed VT events confined to patients with residual or unablated substrate. These findings argue that successful elimination of a SCAI reclassifies the patient from a higher to a lower arrhythmic risk stratum, an effect that no ICD-based strategy can achieve.

Three caveats temper this comparison. First, the published proactive cohorts are short relative to the lifetime risk horizon of a rTOF patient, and durability of bidirectional block beyond five years remains incompletely characterized. Second, the comparison between strategies is necessarily indirect. No randomized trial has compared proactive and reactive approaches head-to-head, and the apparent superiority of the proactive strategy may reflect, at least in part, selection of lower-risk patients into proactive cohorts. Third, the proactive paradigm carries an inherent overtreatment risk: a fraction of patients with identifiable SCAI would never have developed clinical VT, and the procedural risk, however low, is incurred without certain individual benefit.

The most defensible clinical posture, given current evidence, is therefore neither a categorical shift to proactive ablation nor a reflexive adherence to reactive practice, but a deliberate matching of strategy to patient phenotype within a framework of shared decision-making. In patients undergoing planned PVR, particularly transcatheter PVR in the native RVOT, proactive substrate evaluation appears reasonable and may be favored when local expertise is available, both because of the procedural opportunity and because subsequent valve placement may close the therapeutic window for endocardial access to Isthmus 3. The discussion with the patient should frame proactive ablation as a favored option in the pre-PVR setting while acknowledging that prospective validation and long-term durability data are still pending. In patients with established clinical VT, reactive ablation remains the standard of care and is supported by the longest available outcome data. Shared decision-making should be focused on the balance between procedural risk and the durability of arrhythmia control rather than on whether to proceed. The intermediate population, asymptomatic patients with high-risk imaging or electrophysiological features but no planned valve intervention, represents the principal area of unresolved practice. In this group the decision rests on an individualized weighing of substrate findings, lifetime arrhythmic risk, procedural risk, and patient values, ideally within a structured shared decision-making conversation that explicitly acknowledges the evolving evidence base and the potential for overtreatment. The results of CATAPULT-TOF and subsequent prospective work will be most informative for this intermediate group and may eventually narrow the range over which clinical equipoise dictates that the choice be patient-driven rather than guideline-directed.

## 9. Limitations and Unresolved Questions

Several methodological constraints limit the strength of inference that can be drawn from the existing literature on proactive ablation in rTOF. Nearly all published cohorts are observational and single- or limited-multicenter, with retrospective design, non-uniform patient selection, and follow-up durations that are short relative to the lifetime arrhythmic risk horizon of the rTOF population. No completed randomized comparison exists between proactive and reactive strategies, and the only ongoing prospective multicenter trial, CATAPULT-TOF, is restricted to the pre-PVR setting [7]. Indirect comparison across cohorts is further complicated by heterogeneity in ablation modality (catheter vs. surgical, radiofrequency vs. cryothermal), procedural endpoint (bidirectional block, non-inducibility, or both), and definitions of clinical success. The reported absence of recurrence in proactive cohorts must therefore be interpreted in the context of follow-up that has not yet reached the interval at which late substrate evolution typically produces clinical events. Finally, cost-effectiveness has not been formally evaluated, and the optimal integration of substrate ablation with primary-prevention ICD decision-making remains undefined.

Additional limitations arise from the narrative design of this review. Although we sought to synthesize the most relevant mechanistic, observational, and guideline literature, the search was not registered, formal risk-of-bias scoring was not performed, and the manuscript does not provide a quantitative synthesis.

## 10. Proposed Clinical Framework

**Figure 5:**
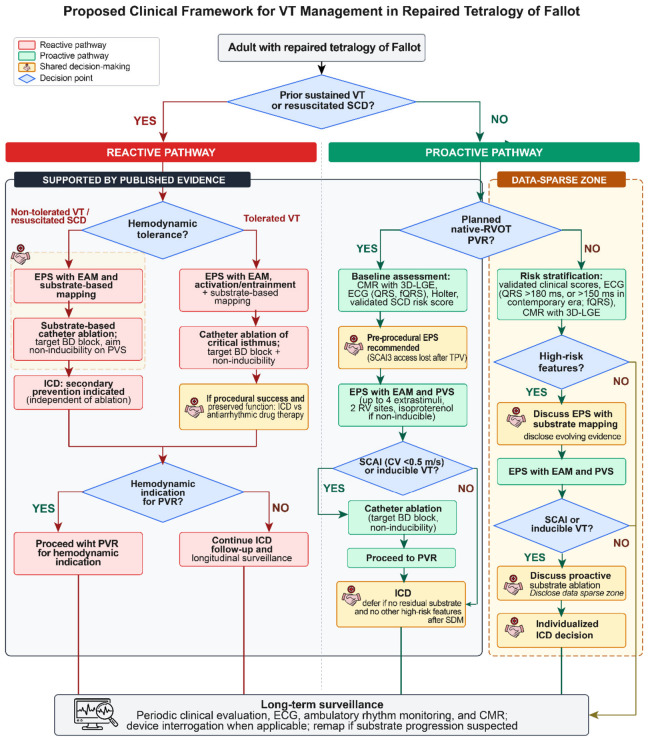
Proposed clinical framework for VT management in adults with repaired tetralogy of Fallot, organized around two entry decisions: a prior history of sustained VT or resuscitated SCD (reactive pathway, red) and the presence of a planned native-RVOT PVR (proactive pathway, green). The reactive pathway is supported by published outcome data; the proactive pathway is supported by published evidence in the pre-PVR setting (solid border) and falls within a data-sparse zone (dashed border) for asymptomatic patients without a planned PVR, in whom decisions are individualized through shared decision-making (handshake icon). Decision points are shown as blue diamonds. The framework is consistent with the 2022 AHA Scientific Statement on arrhythmias in repaired tetralogy of Fallot, the 2018 AHA/ACC guideline for adults with congenital heart disease, the 2019 HRS/EHRA/APHRS/LAHRS expert consensus statement on catheter ablation of ventricular arrhythmias, and the 2022 ESC guidelines on ventricular arrhythmias and prevention of sudden cardiac death, and is intended as a general guide rather than a prescriptive algorithm, applied within shared decision-making and adapted to local expertise, and patient values. Abbreviations: BD, bidirectional; CMR, cardiac magnetic resonance; CV, conduction velocity; EAM, electroanatomical mapping; ECG, electrocardiogram; EPS, electrophysiology study; fQRS, fragmented QRS; ICD, implantable cardioverter-defibrillator; LGE, late gadolinium enhancement; PVR, pulmonary valve replacement; PVS, programmed ventricular stimulation; RV, right ventricle; RVOT, right ventricular outflow tract; SCAI, slowly conducting anatomical isthmus; SCAI3, Isthmus 3; SCD, sudden cardiac death; SDM, shared decision-making; PVR, transcatheter pulmonary valve; VT, ventricular tachycardia.

The strategic considerations developed in the preceding section translate into an operational pathway organized around the patient’s relationship to PVR and to a prior clinical VT event. The framework presented here is consistent with the 2022 AHA Scientific Statement on arrhythmias in repaired tetralogy of Fallot, the 2018 AHA/ACC guideline for adults with congenital heart disease, the 2019 HRS/EHRA/APHRS/LAHRS expert consensus statement on catheter ablation of ventricular arrhythmias, and the 2022 ESC guidelines on ventricular arrhythmias and prevention of SCD [3,9,10,25]. It is intended as a practical decision framework rather than a prescriptive algorithm and is applied within shared decision-making, with adaptation to local expertise, available imaging, and patient values (Figure 5).

### 10.1. rTOF Patients Planned for PVR

Pre-procedural assessment includes 12-lead ECG with attention to QRS duration and fragmentation, ambulatory rhythm monitoring, transthoracic echocardiography, CMR with 3D late gadolinium enhancement when available, and calculation of multiple validated SCD risk scores. Adults referred for native-RVOT PVR may be considered candidates for pre-procedural EPS with electroanatomic mapping, given the inability to access Isthmus 3 once a transcatheter valve is deployed. Mapping characterizes all four anatomical isthmuses with multipolar high-density catheters and quantifies conduction velocity across each; PVS with at least two 8-beat drive trains, up to four extrastimuli, coupling intervals down to ≥180 ms, from at least two right ventricular sites (one near the outlet septum), with isoproterenol challenge if non-inducible at baseline, completes the inducibility assessment. Linear ablation transecting any confirmed SCAI may be reasonable regardless of inducibility, given that the substrate may become inaccessible after valve deployment, with procedural endpoints of bidirectional block confirmed by differential pacing and non-inducibility on repeat stimulation. Post-procedural ICD decision-making follows the 2018 AHA/ACC criteria for primary-prevention ICD in repaired tetralogy of Fallot, integrating left ventricular dysfunction, non-sustained VT, QRS duration, extent of RV scarring, and inducibility on EPS [9]; in patients rendered non-inducible without residual substrate and without other high-risk features, primary-prevention ICD implantation may be deferred after individualized review. Ablation should ideally be performed during the same procedure or the same admission as PVR to preserve catheter access to Isthmus 3.

### 10.2. rTOF Patients Not Planned for PVR

In the absence of a planned valve intervention, the framework rests on non-invasive risk stratification first, with invasive EPS reserved for patients with concerning features. Initial evaluation includes guideline-based clinical risk assessment, 12-lead ECG, ambulatory monitoring, and CMR with 3D-LGE when available. EPS with electroanatomic mapping is reasonable in patients with extensive scar burden on CMR, prolonged or fragmented QRS (recognizing that contemporary surgical eras may produce a substrate at QRS thresholds as low as 150 ms), non-sustained VT, ventricular dysfunction, or a history of sustained supraventricular arrhythmia. When EPS identifies a SCAI or inducible sustained VT, proactive ablation may be offered following an explicit shared decision-making discussion that addresses procedural risk, residual uncertainty about long-term durability, and the possibility that the substrate would never have produced a clinical event. ICD decision-making in this group parallels the post-ablation result and follows the 2018 AHA/ACC primary-prevention criteria, with substrate findings informing rather than replacing the conventional risk-score framework.

### 10.3. After Clinical VT (Reactive Setting)

Two clinical phenotypes warrant separate consideration. In patients with hemodynamically non-tolerated VT or resuscitated sudden cardiac arrest, secondary-prevention ICD implantation is indicated independent of ablation outcome; EPS with electroanatomic mapping and substrate-based ablation, with procedural endpoints of bidirectional block and non-inducibility, addresses arrhythmia burden and reduces appropriate shock frequency but does not obviate device implantation. In patients with hemodynamically tolerated VT, particularly those with preserved ventricular function and an absence of other high-risk features, current expert consensus acknowledges that successful catheter ablation, defined by complete bidirectional block across all critical isthmuses and non-inducibility on repeat stimulation, may serve as an alternative to ICD therapy after explicit shared decision-making, given that fewer than 15 percent of patients referred for VT ablation have reduced left ventricular systolic function and long-term VT-free survival in this subgroup is favorable [4]. When VT is hemodynamically tolerated and inducible, activation and entrainment mapping confirm the critical isthmus; when VT is non-tolerated or non-inducible, substrate-based ablation targets identified SCAI. PVR in patients with prior clinical VT is performed for hemodynamic rather than antiarrhythmic indications; concomitant surgical ablation may be considered when surgical PVR is undertaken in patients with mapped substrate that has not been addressed by prior catheter ablation. Post-procedural surveillance includes device interrogation when applicable, periodic ambulatory monitoring, and repeat imaging to detect substrate progression that may warrant remapping.

## 11. Future Directions

We propose the following future directions, organized in descending order of impact and feasibility (Table 3): First and most consequentially, prospective multicenter validation of proactive ablation beyond the pre-PVR setting is the central evidence gap. CATAPULT-TOF will deliver the first prospective dataset within that window [7], but high-risk asymptomatic adults without planned PVR, patients referred for atrial arrhythmia ablation, and adolescents with early-identifiable Isthmus 3 require dedicated cohorts. Pragmatic registry-based designs through international consortia will likely be the most realistic vehicle. Second, standardization of procedural endpoints, ablation protocols, and outcome reporting across centers is essential before comparative-effectiveness analyses become meaningful. Common data elements developed under professional-society leadership would accelerate evidence generation more than any single trial. Third, formal cost-effectiveness modeling, integrating the avoided downstream costs of ICD complications against the upfront costs of mapping and ablation, will determine whether health systems can scale this strategy. Without these analyses, adoption will remain confined to centers willing to absorb resource intensity that has not been formally justified. Fourth, artificial intelligence applied to the ECG and to 3D LGE-CMR is positioned to reshape risk stratification. AI-ECG models may detect substrate-suggestive features that escape visual interpretation, and convolutional networks applied to 3D LGE-CMR are approaching automated SCAI delineation. If prospectively validated, these tools could focus invasive EAM on the highest-yield patients and extend proactive evaluation to centers without specialized congenital electrophysiology expertise. Fifth, patient-reported outcomes, including device-related anxiety, body image, and quality of life, should be incorporated as primary endpoints in prospective studies, alongside individualized decision aids that quantify ablation benefit, ICD avoidance, and procedural risk. The strongest argument for the proactive paradigm will be made when it delivers not only fewer arrhythmic events but also less device-dependent lives. Looking further ahead, several technological advancements will likely reshape the field over the next decade: pulsed-field ablation as a tissue-selective alternative for right ventricular substrates, stereotactic body radiotherapy as a potential salvage strategy for substrates rendered inaccessible after PVR deployment, patient-specific computational models that allow virtual testing of ablation strategies before catheter delivery, and polygenic risk scores integrated with circulating fibrosis biomarkers to add a molecular layer to risk projection. Last, and easily underweighted, is the equity of access. High-quality rTOF VT ablation is concentrated in a small number of academic centers, and a paradigm shift toward proactive intervention risks widening existing disparities in adult congenital heart disease care unless deliberate effort expands training, broadens geographic access, and integrates non-tertiary centers into the referral and follow-up infrastructure. The technical advances above will matter only insofar as they reach the patients who need them.

## 12. Conclusions

Catheter ablation of the SCAI has matured from a salvage therapy after clinical ventricular tachycardia into a substrate-directed strategy that can, in selected settings, be deployed before the first arrhythmic event. The accumulating observational evidence suggests that proactive ablation is feasible and safe and may reduce subsequent primary-prevention defibrillator need in carefully selected patients, particularly when performed before native-RVOT transcatheter PVR. However, whether proactive ablation should displace reactive practice across the broader rTOF population awaits prospective data from CATAPULT-TOF [7] and subsequent multicenter studies. The pre-PVR setting offers a potential indication, although prospective validation is still pending, because procedural opportunity, risk of losing endocardial access to Isthmus 3, and supporting observational evidence converge. The principal task ahead is to define the population in whom proactive substrate evaluation should become routine rather than selective, while ensuring that ablation refines but does not automatically replace current standard of care (i.e., ICD implantation) in patients with persistent high-risk features.

## Figures and Tables

**Table 1:** Shared decision-making considerations for proactive ablation outside the pre-PVR setting.

Domain	Potential Risk or Uncertainty	How to Discuss with the Patient
Substrate elimination before first clinical VT	No prospective proof that elective ablation improves survival or event-free survival in non-PVR asymptomatic patients	Frame benefit as plausible but unproven outside the pre-PVR window
Possible reduction in future ICD candidacy	Ablation does not protect against polymorphic VT/VF or risk driven by ventricular dysfunction, extensive fibrosis, or residual substrate	Clarify that ablation may refine, not replace, ICD decision-making
Use of an already planned invasive EP procedure, such as atrial arrhythmia ablation	Incremental mapping and ablation may prolong the procedure and add vascular, perforation, or conduction-system risk	Balance incremental risk against the value of obtaining substrate information
Avoiding loss of an opportunity if substrate later becomes inaccessible	In patients without planned PVR, the access-related rationale is weaker than in the pre-PVR setting	Explain why timing is less urgent than before native-RVOT transcatheter PVR
Psychological benefit from a proactive plan	Overtreatment of a SCAI that may never have produced clinical VT	Include patient preferences, anxiety, willingness for surveillance, and tolerance of procedural risk

**Table 2:** Reactive vs. proactive VT ablation in rTOF: a comparative framework. Reference numbers correspond to the manuscript bibliography. The proactive evidence base derives almost entirely from cohorts in which ablation was performed at, or in anticipation of, PVR; data on stand-alone elective proactive ablation in patients without a planned valve intervention remains limited. Abbreviations: CMR, cardiac magnetic resonance; EAM, electroanatomical mapping; EPS, electrophysiology study; HR, hazard ratio; ICD, implantable cardioverter defibrillator; LVEF, left ventricular ejection fraction; PVR, pulmonary valve replacement; PVS, programmed ventricular stimulation; RR, relative risk; RVOT, right ventricular outflow tract; SCAI, slowly conducting anatomical isthmus; SCD, sudden cardiac death; PVR, transcatheter pulmonary valve; VT, ventricular tachycardia.

Domain	Reactive Ablation	Proactive (Prophylactic) Ablation
Definition and trigger	Substrate- or VT-targeted ablation in response to documented sustained VT, VT storm, or recurrent appropriate ICD therapies.	Substrate-based EAM-guided SCAI ablation in the absence of prior sustained VT, typically at or before planned PVR or in selected high-risk asymptomatic patients.
Target population	Patients with documented clinical VT or appropriate ICD therapy.	Patients without prior clinical VT, identified through pre-PVR EPS, CMR, or clinical risk stratification.
Timing relative to substrate	Late, after substrate has matured and supported clinical reentry.	Early, while substrate is identifiable but not yet supporting sustained VT; preserves access to Isthmus 3 before native-RVOT PVR.
Procedural objective	Transection of VT-critical isthmus and/or all SCAIs; non-inducibility on repeat PVS.	Transection of all identified SCAIs regardless of inducibility; non-inducibility on repeat PVS.
Acute procedural success	82% acute success in the largest long-term series (n = 34) [12]; 91% acute non-inducibility in foundational mapping cohort (n = 11) [11].	87% complete SCAI transection in the largest contemporary single-center cohort; and higher success rate in selected smaller series [28,38].
Long-term arrhythmia outcomes	Freedom from death and arrhythmia recurrence 94%/81%/70% at 5/10/20 years; meta-analysis RR 0.11 for VT recurrence with SCAI-based ablation [12,27].	0% sustained VT in patients with successful SCAI transection over median 21- to 58-month follow-up; events confined to patients with residual or unablated SCAI [28,38].
Procedural safety	Major complication rate 5–10% in pooled data, predominantly vascular access; cardiac perforation and complete heart block uncommon; no procedure-related deaths reported [27].	No ablation-related complications reported across the largest contemporary cohorts (n = 57 and n = 97) [28,38].
Impact on ICD implantation	Patients are typically already ICD-protected (secondary prevention); ablation reduces shock burden, but ICD remains in place.	Reduces primary-prevention ICD candidacy from 25 to 51% (depending on guideline applied) to 11%; ICD deferred in most patients with successful substrate elimination [28,38].
SCD prevention strategy	Relies on ICD as safety net; residual annual appropriate shock rate 7.7–9.8% in primary/secondary prevention cohorts [21].	Substrate elimination without device dependency in selected patients with successful SCAI transection; no SCD reported in published proactive cohorts to date, but follow-up remains short.
Quality of evidence	Observational cohorts and one meta-analysis spanning > 15 years; consistent across centers.	Modest-strength evidence: two single-center retrospective comparisons, one mixed pre-/peri-PVR cohort, and one ongoing prospective multicenter trial (CATAPULT-TOF); no complete randomized comparison [7,28,38].
Principal limitations	Late intervention; ongoing substrate evolution after index ablation; LVEF < 60% predicts recurrence despite successful ablation (HR 16.4); persistent need for ICD backup.	Risk of overtreatment in patients who would never have manifested clinical VT; short follow-up; selection bias, single-center and historical-control designs; cost-effectiveness undefined; durability of bidirectional block beyond 5 years not yet established.

**Table 3:** Future directions in proactive vs. reactive VT management in repaired tetralogy of Fallot. Rows are ordered by descending priority on the basis of anticipated impact and feasibility. Directions 1–5 represent near- and mid-term priorities most directly bearing on clinical practice. Directions 6–10 represent technological and biological lines that are likely to reshape the field over a longer horizon. The final entry (equity of access) is foundational to the population-level impact of all preceding directions. Abbreviations: ACHD, adult congenital heart disease; AI, artificial intelligence; CMR, cardiac magnetic resonance; EAM, electroanatomical mapping; EHRA, European Heart Rhythm Association; EP, electrophysiology; HRS, Heart Rhythm Society; hs-troponin, high-sensitivity troponin; ICD, implantable cardioverter defibrillator; ISACHD, International Society for Adult Congenital Heart Disease; LGE, late gadolinium enhancement; NT-proBNP, N-terminal pro B type natriuretic peptide; PACES, Pediatric and Congenital Electrophysiology Society; PFA, pulsed-field ablation; PVR, pulmonary valve replacement; rTOF, repaired tetralogy of Fallot; SBRT, stereotactic body radiotherapy; SCAI, slowly conducting anatomical isthmus; sST2, soluble suppression of tumorigenicity 2; PVR, transcatheter pulmonary valve; VT, ventricular tachycardia.

Research Direction	Specific Aims	Current State	Methodological Approach	Anticipated Impact
Prospective validation of proactive ablation beyond pre-PVR setting	Outcomes of proactive SCAI ablation in (a) high-risk asymptomatic patients without planned PVR, (b) patients undergoing atrial arrhythmia ablation, (c) adolescents with early-identifiable Isthmus 3	CATAPULT-TOF will provide the first prospective multicenter dataset within the pre-PVR setting; non-PVR populations remain unstudied	Pragmatic registry-based or stepped-wedge designs through international congenital EP consortia	Defines whether the proactive paradigm extends beyond the peri-PVR window; foundation for guideline revision
Standardization of procedural protocols and outcome reporting	Define common data elements for substrate definition, ablation endpoints, follow-up intervals, and event adjudication	Heterogeneity in inducibility protocols, ablation modality, and endpoint definitions limits cross-cohort comparability	Professional-society-led consensus initiatives (PACES, HRS, EHRA, ISACHD) with harmonized core data dictionary	Enables meaningful meta-analysis and accelerates evidence generation across centers
Cost-effectiveness and resource-utilization modeling	Quantify net cost of proactive vs. reactive strategy, integrating ICD avoidance, ablation cost, and downstream complications	No formal cost-effectiveness analysis has been published for either strategy	Markov modeling using published event rates, complication probabilities, and quality-adjusted life-year estimates	Determines feasibility of system-level adoption; guides payer and health-system decisions
Artificial intelligence applied to ECG and CMR	AI-ECG detection of substrate-suggestive features; automated SCAI delineation on 3D LGE-CMR; AI-driven multimodal risk score	Early models report > 90% sensitivity for AI-CMR SCAI detection; AI-ECG models in development	Multicenter labeled training datasets; prospective validation against EAM-defined substrate; external generalizability testing	Non-invasive screening would focus invasive EAM on highest-yield patients and democratize substrate evaluation beyond expert centers
Patient-reported outcomes and decision aids	Quantify device-related anxiety, body-image impact, exercise behavior, and quality of life in proactive vs. reactive strategies; develop a validated decision aid for shared decision-making	Patient-reported endpoints rarely incorporated as primary outcomes in the rTOF VT literature	Embedded patient-reported outcome instruments in prospective cohorts; co-design of decision aid with patient stakeholders	Strengthens the case for the proactive strategy on patient-centered grounds; harmonizes shared decision-making across centers
Pulsed-field ablation in rTOF substrate	Safety, efficacy, and durability of PFA for SCAI transection compared with radiofrequency; performance in the presence of nitinol valve frames	Investigational in the right ventricle; published reports of voltage-gradient disruption by nitinol limit current applicability	Phase I/II safety studies in rTOF cohorts; bench characterization of PFA in PVR-adjacent substrate	Tissue-selective alternative may reduce collateral injury risk and enable post-PVR ablation if voltage-shielding limitations are addressed
Stereotactic body radiotherapy as post-PVR salvage	Determine whether SBRT can deliver durable substrate modification when endocardial access is lost after PVR deployment	Limited spatial precision adjacent to the conduction system; reported semilunar valve injury constrains application	Dose-escalation studies; integration with cardiac MRI for target delineation; small prospective cohorts	A viable post-PVR strategy would meaningfully reduce the urgency of pre-PVR ablation and expand options for patients with established substrate after valve placement
Patient-specific computational electrophysiology	Develop digital-twin models integrating individual anatomy, scar distribution, and electrophysiological properties; virtual ablation planning	Proof-of-concept demonstrations; prospective clinical validation lacking	Image-derived mesh models; in silico simulation of reentry circuits; comparison with invasive EAM	Pre-procedural ablation planning may reduce procedure duration, improve substrate transection, and individualize strategy
Genomic and biomarker-based risk refinement	Contribution of channelopathy variants and polygenic risk to arrhythmic risk; role of fibrosis biomarkers (galectin-3, sST2) and ventricular stress markers (NT-proBNP, hs-troponin)	Coexisting channelopathy variants identified in case series; biomarker integration largely uncharacterized	Genome-wide and targeted sequencing in defined rTOF cohorts; biobank-linked biomarker analyses with prospective event capture	Adds molecular dimension to multimodal risk projection beyond clinical, imaging, and electrophysiological data
Longitudinal substrate surveillance	Define optimal frequency and triggers for repeat non-invasive imaging and invasive mapping; integrate continuous wearable rhythm monitoring	A single cross-sectional EAM may miss continuous substrate evolution; surveillance intervals undefined	Prospective serial imaging cohorts; integration of multi-week wearable ECG monitoring; analysis of interval-change predictors	Reframes substrate evaluation from static events to dynamic surveillance; identifies the optimal moment for re-intervention
Equity of access and training capacity	Geographic and demographic distribution of high-volume rTOF VT ablation; training pipeline for ACHD-electrophysiology dual expertise; integration of non-tertiary centers into referral networks	Capability concentrated in a small number of academic centers, raising risk of widening disparities	Workforce surveys; regional capacity analyses; structured fellowship pathways; tele-EP collaboration models	Determines whether technical advances reach the patients who need them; foundational to translating any of the directions above into population-level benefit

## Data Availability

No new data were created or analyzed in this review. Data sharing is not applicable.

## Abbreviations

TOF	Tetralogy of Fallot
AI	Anatomical Isthmus
SCAI	Slow-Conducting Anatomical Isthmus
EPS	Electrophysiology Study
VT	Ventricular Tachycardia
NSVT	Non-Sustained Ventricular Tachycardia
RV	Right Ventricle
LV	Left Ventricle
PVR	Pulmonary Valve Replacement
EAM	Electroanatomical Mapping
CV	Conduction Velocity
LGE-CMR	Late Gadolinium Enhancement-Cardiac Magnetic Resonance
SCD	Sudden Cardiac Death
ILAM	Isochronal Late Activation Mapping
NPV	Negative Predictive Value
PPV	Positive Predictive Value
WCD	Wearable Cardioverter-Defibrillator
PVS	Programmed Ventricular Stimulation
